# The Phenolic Composition, Antioxidant Activity and Microflora of Wild Elderberry in Asturias (Northern Spain): An Untapped Resource of Great Interest

**DOI:** 10.3390/antiox12111986

**Published:** 2023-11-09

**Authors:** Roberto Rodríguez Madrera, Rosa Pando Bedriñana

**Affiliations:** Área de Tecnología de los Alimentos, Servicio Regional de Investigación y Desarrollo Agroalimentario (SERIDA), E-33300 Villaviciosa, Spain; rpando@serida.org

**Keywords:** elderberry, polyphenols, anthocyanin, flavonols, antioxidant activity, microflora

## Abstract

The objective of this study is the characterization of the phenolic profile and antioxidant activity of elderberries (*Sambucus nigra* L.) from a collection of 79 wild specimens in northern Spain to assess variations in the species at the local level and evaluate its interest as a source of biocompounds. Also, a first study was carried out on the microflora present in this fruit, providing information relevant to its commercial exploitation. Moreover, the phenolic composition, antioxidant capacity and microbial composition in overripe fruits were determined, seeking a better use for this currently wasted resource. A wide variability in levels of phenolics was detected. Elderberries showed high antioxidant activity related to a high cyanidin derivative content, making them of interest to industry. Microflorae were present in very variable concentration ranges, so their levels should be monitored in those applications that require strict control. Overripe fruits are of interest as a source of anthocyanidins, since their concentration and antioxidant capacity remain after the optimal ripening period, promoting sustainability and a better use of natural resources. The database generated is of particular interest for further breeding trials based on the phenolic profile and antioxidant activity of the samples.

## 1. Introduction

European elderberry (*Sambucus nigra* L.) is a species of the Sambucus genus, belonging to the Caprifoliaceae family. *S. nigra* is a shrub widely distributed in Europe, North America, East Asia and North Africa in the wild and whose cultivation is expanding [1,2]. The fruits are globous berries, small, black and rich in bioactive, functional and nutritional compounds such as sugars, organic acids, phenolic compounds, pectin, fatty acids and minerals [3,4,5,6].

The fruit, leaves and flowers of *S. nigra* have been widely used in folk medicine in Europe to treat cutaneous, diuretic and viral infections, constipation, colds, flu, catarrh, influenza, inflammation, joint pain, fever, respiratory disturbances, sciatica, headache, dental pain, heart pain and nerve pain [2,7,8], and nowadays, remedies against colds, flu and other infectious illnesses are being developed using *S. nigra* fruit or flowers [9].

In its report EMA/HMPC/44208/2012, the European Medicines Agency [7] notes that the fruit of *S. nigra* contains several components that may contribute to pharmacological activity, with various phenolic compounds among them, such as anthocyanins and flavonols, possessing notable antioxidant activity. However, the concentration of polyphenols varies depending on several factors such as the genotype, the environmental conditions or the degree of ripening of the fruit [3,10,11,12,13,14,15], so the content of this group of nutraceuticals shows wide variability. Thus, differences of more than 100% have been reported in the content of the different phenolic compounds for varieties in the same location [3,5]. It has also been proven that, in the ripe fruit, anthocyanins are the main extractable phenolic compounds, while in the green fruit, this family of flavonoids is not present, and flavonols and phenolic acids predominate [13,16,17]. Imenšek et al. [13] have shown that when the fruits are overripe (more than 90% of the fruits are shrivelled due to moisture loss) the nutritional quality (sugars and organic acids) of elderberries decreases, which is why the authors proposed harvesting the fruit when it is completely ripe or even before the complete maturation of all the berries in the bunch. Nevertheless, when the fruits are overripe, the antioxidant activity is equal to or greater than that of the fully matured fruit [13], so overripe fruits could be an affordable source of bioactive compounds for diverse applications such as cosmetics, pharmaceuticals or agriculture [9,18,19]. In this sense, the efficient use of resources and the application of solutions based on natural products provide environmental, social and economic benefits.

On the other hand, it has been reported that in grapes, the loss of moisture during overripeness and breakage favour the presence of microorganisms such as yeasts, bacteria and fungi, affecting the quality of the fruit, fruit juices and wines. These microorganisms can cause the spoilage of the smell and taste, leading to economic losses. Additionally, if the spoiling fungi are producers of mycotoxins or pathogenic, they could pose a health risk to consumers [20,21]. Therefore, using a raw material with a low microbial load is important, as it facilitates fruit conservation and/or drying processes, favours the stability of the juices and prevents alterations during fermentation processes. However, as far as we know, there is no information on the microflora present in elderberries during the ripening period, which is undoubtedly of interest for the different applications of the fruit.

Although the growing interest in the fruits and flowers of *S. nigra* has led to its cultivation being promoted in different parts of Europe, with cultivars well adapted to local conditions being available, the truth is that elderberry is a mainly found as a wild plant [22]. In the specific case of Spain, where wild specimens are widespread in the northern and western part of the territory [1], the use of the flowers and fruits of *S. nigra* is practically null, being limited to the domestic preparation of folk medicine remedies or products such as jams, jellies and desserts, with no commercial utilization of the plant. However, both for its health benefits [23] and for different applications in the food industry [24], elderberry is a non-timber forest species whose exploitation is undoubtedly of interest and may be viable, given its good adaptation and expansion throughout the territory. In this regard, it should be noted that elderberry cultivation has been successfully established in the Iberian Peninsula at Varosa Valley (Northern Portugal) for decades [10]. However, there are no data on local specimens, which are of great interest due to their capacity to adapt to local conditions.

Given the interest of elderberry products for their nutraceutical components, and the large number of factors that influence their composition (genotype, climate, soil, degree of maturation), the objective of this study is the characterization of the phenolic profile and antioxidant activity in wild specimens of *S. nigra* in Asturias (northern Spain), in order to assess the variation in these characteristics in the species on a local level and evaluate the interest of individual trees as a source of biocompounds. Also, a first study was carried out on the microflora present in the fruit, which will provide information of interest for establishing the most appropriate uses of the fruit. Likewise, the phenolic composition, antioxidant capacity and microbial composition of overripe fruits were determined, looking for a better use of this currently wasted resource.

## 2. Materials and Methods

### 2.1. Reagents and Standards

ABTS (2,2-azino-bis-3-ethylbenzothiazoline-6-sulfonic acid), DPPH (2,2′-diphenyl-1-picrylhydrazyl), Trolox (6-hydroxy-2,5,7,8-tetramethylchroman-2-carboxylic acid), potassium persulphate, gallic acid, neochlorogenic acid and sodium acetate were purchased from Sigma Aldrich. Quercetin, quercetin 3-*O*-glucoside, kaempferol 3-glucoside, quercetin 3-*O*-rutinoside, kaempferol 3-*O*-rutinoside, isorhamnetin 3-*O*-glucoside, isorhamnetin 3-*O*-rutinoside, pelargonidin 3-*O*-glucoside, cyanidin 3-*O*-glucoside, cyanidin 3,5-*O*-diglucoside, cyanidin 3-*O*-sambubioside-5-*O*-glucoside and chlorogenic acid were purchased from Extrasynthèse (Genay, France). Cyanidin-3-*O*-rutinoside and cyanidin 3-O-sambubioside were purchased from Biosynth International (San Diego, CA, USA) and cryptochlorogenic acid was purchased from Purifa-Cymit (Barcelona, Spain). Water was purified using a Milli-Q system from Millipore (Bedford, MA, USA). Solvents were purchased from Panreac (Barcelona, Spain) and were of analytical or HPLC grade.

### 2.2. Plant Material

**Figure 1:**
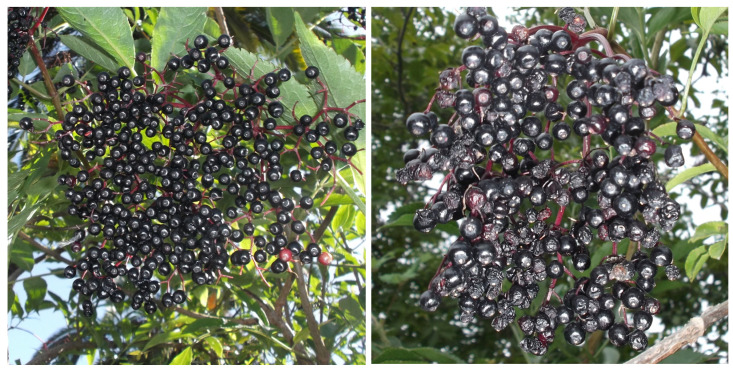
Fully ripe (**left**) and overripe (**right**) elderberries.

The fruits of 79 wild specimens of elderberry (*S. nigra* L.) were collected in different locations of Asturias (Northern Spain) in 2021, 2022 and 2023 (Supplementary Table S1). The samples (200–250 g) were taken during the months of July to September. The whole clusters were harvested when fully ripe (FR) according to Imenšek et al. [25]; that is, the exterior of all berries exhibited the final black colour (Figure 1). Once in the laboratory, the berries were separated from the stems, frozen, lyophilized and stored under vacuum conditions at −20 °C and protected from light. These samples were used to establish the phenolic content and antioxidant capacity of the species at a local level.

For another series of experiments, elderberry samples were taken, during 2022 and 2023, from 24 individuals in two stages of maturation: FR (defined previously) and overripe (OR); that is, when around 25–50% of the fruits were shrivelled due to the loss of moisture (Figure 1). From the elderberry samples, collected in sterile containers with screw caps and transported promptly to the laboratory, an aliquot of 10 g of stemless elderberries was used to carry out the microbiological analyses. The rest were preserved, as explained above, to determine the phenolic content and antioxidant capacity.

Moreover, to provide a source of comparison with the local wild elderberries, different berries characterized by their high polyphenol content and good antioxidant activity were analysed. These other berries were a cultivar of blackberry (*cv* Prime-Arc^®^ 45), a cultivar of raspberry (*cv* Versalles) and three cultivars of blueberry belonging to the species *Vaccinium corymbosum* (*cv* Bluecrop), *V. virgatum* (*cv* Alapaha) and a *V. sp.* hybrid (*cv* Northblue), previously freeze-dried, as well as a commercial sample of dried elderberry.

### 2.3. Phenolic Extracts

At the moment of the chemical analysis, the lyophilized elderberries were ground in a mortar and sieved through a standard sieve (pore size 1.00 mm).

The extraction of polyphenols and HPLC analysis were carried out according to a previously validated method with slight modifications [26]. Briefly, 0.1 g of freeze-dried elderberry powder was mixed with 30 mL of 46% aqueous ethanol (0.1% perchloric acid) over a period of 10.3 min in a water bath at 20 °C, using an ultrasonic homogenizer UP200Ht (Hielscher, Teltow, Germany) equipped with a 2 mm diameter sonotrode at a frequency of 26 kHz. After extraction, the solids were separated via centrifugation; the supernatant was dried in a rotary vacuum evaporator at 40 °C, after which the residue was reconstituted with 10 mL of 20% aqueous methanol (0.1% perchloric acid) and filtered through a 0.45 μm PVDF syringe filter (Teknokroma, Barcelona, Spain). The extractions were carried out in duplicate. These extracts were used to perform the assays described in Section 2.4 and Section 2.5.

### 2.4. Phenolic Composition

#### 2.4.1. HPLC Analysis

Eighteen phenolic compounds were analysed in the extracts: 3 caffeoylquinic acids, 7 anthocyanins and 8 flavonols. Analyses were performed with a Waters system equipped with a 717 plus autosampler, a temperature controller, a model 600 Controller pump, a DAD 2996 diode array detector and using the Empower v.3.0 data module software (Waters Associates, Milford, MA, USA). The separation of polyphenols was carried out in a reverse-phase Macherey-Nagel Nucleosil 120 C_18_ column (250 × 4.6 mm I.D, 3 μm) from Fisher Scientific (Leicestershire, UK). The column was thermostated at 45 °C and the injection volume was 50 µL. The elution solvents were aqueous 2% acetic acid (solvent A) and 100% methanol (solvent B). The samples were eluted at a flow rate of 1.0 mL/min according to the following conditions: linear gradient from 0 to 20% B until min 37, isocratic until min 60, linear gradient to 45% B until min 80, linear gradient to 55% B until min 85, linear gradient to 65% B until min 95, linear gradient to 0% B until min 105 and isocratic until min 125.

MS spectra were recorded using an Agilent 1290 Infinity LC System equipped with an Agilent 6470A triple quadrupole using the separation conditions described above. The mass spectrometer operated in negative and positive ionization modes and the spectra were recorded by scanning the mass range from *m*/*z* 50 to 1000. Nitrogen was used as the drying, nebulizing and collision gas. The drying gas flow was 12 L/min at 350 °C. The nebulizer pressure was 50 psi and the capillary voltages were 4000 V and 3500 V in the positive and negative ionization modes, respectively. For the tandem MS (MS2), the collision energy was set at 30 eV. The identity of the polyphenols was ascertained using data from the DAD and MS analysis, through a comparison and combination of their retention times, UV–vis and mass spectra, and confirmed with authentic standards when available.

Quantitation was performed at 313 nm (caffeoylquinic acids), 355 nm (flavonols) and 510 nm (anthocyanins), according to an external standard method. For the compounds lacking standards or those for which the amount at our disposal was too small, quantification was achieved from similar compounds. Thus, the caffeoylquinic acids were quantified as chlorogenic acid, anthocyanins as cyanidin 3-*O*-glucoside, quercetin 3-*O*-acetylglucoside as quercetin 3-*O*-glucoside and isorhamnetin derivatives as isorhamnetin 3-rutinoside.

#### 2.4.2. Total Phenolic Content (TPC)

The total phenolic content (TPC) was determined via spectrophotometry using the Folin–Ciocalteu method. The reaction was conducted in 10 mL volumetric flasks, to which the various reactants were added in the following order: 200 µL of appropriately diluted phenolic extract, 5 mL of water, 250 µL of Folin–Ciocalteu reagent, 750 µL of 20% sodium carbonate and water to reach the final volume. The absorbance was measured at 700 nm, after 30 min at room temperature. Gallic acid was used as the standard for the quantification of the total phenolic compounds. The results are expressed as mg of gallic acid equivalent (GAE)/g of dry weight (DW). All extracts were analysed in triplicate.

#### 2.4.3. Monomeric Anthocyanin Content (MAC)

The MAC was determined using the differential pH method described by Wrolstad [27]. Two aliquots of 500 µL of appropriately diluted phenolic extracts were diluted 1/5. One aliquot was diluted with a pH 1.0 buffer (0.025 M potassium chloride) and the other was diluted with a pH 4.5 buffer (0.4 M sodium acetate). Absorbance was measured at 510 nm and 700 nm using a Perkin–Elmer Lambda 35 UV spectrophotometer (Boston, MA, USA). The MAC in the extracts was calculated according to the following formula:

MAC *=* [(A _λ500_ − A _λ700_)_pH1.0_ − (A _λ500_ − A _λ700_)_pH4.5_] × 449.2 × 1000/(26,900 × DF)

where A is the absorbance, 449.2 is the molecular mass of cyanidin-3-O-glucoside, 26,900 is its molar absorptivity (ε) and DF is the dilution factor. The results were expressed as mg of cyanidin-3-O-glucoside equivalent (C3G)/g of DW. All extracts were analysed in triplicate.

### 2.5. Antioxidant Activity

#### 2.5.1. Reducing Power

Reducing power determination was carried out using the FRAP method, according to Benzie and Strain [28]. A working FRAP reagent was prepared daily from the following three solutions in the ratio 10:1:1: 300 mM of acetate, pH 3.6; 10 mM of TPZ (2,4,6-tripyridyl-s-triazine) in 40 mM of HCl and 20 mM of FeCl_3_·6H_2_O. Briefly, 100 µL of appropriately diluted phenolic extracts were mixed with 3.0 mL of the working FRAP reagent in a test tube; the absorbance was read at 593 nm against a reagent blank after 20 min at room temperature. FeSO_4_·7H_2_O solutions were used to construct a standard curve and the results were expressed as µmol of Fe^2+^/g of DW. All extracts were analysed in triplicate.

#### 2.5.2. Radical Scavenging Activity

##### DPPH Assay

The radical scavenging activity was determined using the DPPH method according to Diñeiro García et al. [29]. Forty µL of either the appropriately diluted phenolic extract, the standard (Trolox) or methanol in the case of the reagent blank, were added to 1.460 mL of the DPPH solution (1 × 10^−4^ M) in methanol. Absorbance at 515 nm was measured after 120 min at room temperature when the reaction reached its stable state. The inhibition percentage (IP) was calculated as follows:

IP = [(A_blank_ − A_sample_)/A_blank_] × 100

where A_sample_ is the absorbance of the phenolic extract and A_blank_ is the absorbance of the DPPH solution when methanol is added rather than the sample. Trolox solutions were used to construct a standard curve and the results were expressed as µmol of Trolox equivalent (TE)/g of DW. All extracts were analysed in triplicate.

##### ABTS Assay

A solution of ABTS radical was prepared by mixing (1:1) a 7 mM ABTS stock solution (dissolved in a 20 mM acetate buffer, pH 4.5) with 2.45 of mM potassium persulphate according to Orzan et al. [30]. This mixture was allowed to stand for 12–16 h at room temperature in the dark until reaching a stable oxidative state. It was then diluted in an acidic medium of 20 mM of sodium acetate buffer (pH 4.5) to an absorbance of 0.700 ± 0.010 at 734 nm. Ten µL of phenolic extract, the standard (Trolox) or methanol in the case of the reagent blank, were added to 3 mL of the ABTS solution. Absorbance at 734 nm was measured after 120 min at room temperature. The inhibition percentage (IP) was calculated as described for the DPPH method. Trolox solutions were used to construct a standard curve and the results were expressed as µmol of Trolox equivalent (TE)/g of DW. All extracts were analysed in triplicate.

### 2.6. Microbiological Assays

Ten grams of each elderberry sample was diluted in 90 mL of Ringer solution and homogenized for 2 min at normal speed in a stomacher. Serial dilutions of the suspension were made in the Ringer solution and plated on the surface of an appropriate media. The numbers of microorganisms were expressed in colony-forming units per gram of elderberry (cfu/g). The microbiological analyses included the determination of the total number of mesophilic bacteria, fungi and yeasts, and lactic and acetic bacteria. 

Conditions for microbiological analyses:

− Total number of aerobic mesophilic microorganisms on PCA agar, incubation at 37 °C for 24–48 h;
− Total number of moulds and yeasts on Sabouraud Chloramphenicol Agar (Sharlau), incubation at 25 °C for 24–48 h;
− Total number of lactic acid bacteria (LAB) on MRS agar with pimaricin (50 mg/L) and streptomycin (100 mg/L) at 30 °C for 48–96 h;
− Total number of acetic acid bacteria (AAB) on GYC medium [31] with pimaricin (50 mg/L) and penicillin (25 mg/L) at 30 °C for 72–96 h.

### 2.7. Statistical Analysis

A one-way analysis of variance was carried out to detect significant differences in the composition of elderberries according to the harvest year. A one-way analysis of variance was carried out to detect significant differences in the composition and microbial counts of elderberries and their chemical composition according to the ripening stage. Pearson’s correlation coefficient (r) was computed to estimate correlations between variables and multiple linear regression was used to estimate the antioxidant activities from the components of the phenolic profile of elderberry fruit. The program used was SPSS version 15.0 (SPSS Inc., Chicago, IL, USA).

## 3. Results and Discussion

### 3.1. Phenolic and Antioxidant Characterization of Locally Collected Samples

#### 3.1.1. Phenolic Composition 

The total phenolic content (TPC) and monomeric anthocyanin content (MAC) from each of the sampled individuals are shown in Supplementary Table S1. Table 1 shows the averages of the TPC and MAC for each harvest year.

The TPC contents of the collection were within a wide range, from 24.0 to 76.1 mg of gallic acid equivalents (GAEs)/g of dry weight (DW), with an average of 43.8 mg of gallic acid/g of DW, in accordance with the values reported by Goud et al. [32]. The values present in the literature for the fresh weight (FW) also show wide ranges of variability between 277 mg of GAE/100 g of FW [33] and 1476 mg of GAE/100 g of FW [10], and its content can be considered equivalent to our experimental data if the usual water content in the fresh samples (71–78%) is taken into account [10,34].

Likewise, in the MAC, a notable level of variability was detected, with values covering almost an order of magnitude (6.3–58.3 mg cyanidin 3-*O*-glucoside (C3G)/g DW) and an average value of 26.5 mg of C3G/g of DW. Imenšek et al. [13] found a maximum concentration of MAC of 1156 mg of C3G/100 g of DW at the optimal moment of ripening, a value within the lower range obtained in this study. The measurements reported by other authors also show high variability, with values between 277 mg of C3G/100 g of FW [33] and 1265 C3G mg/100 g of FW [5]. Anthocyanins are the most quantitatively important family of elderberry polyphenols, as evidenced by the high correlation between the TPC and MAC (r = 0.92), with an average value for the MAC/TPC ratio of 0.59, in accordance with the results of other authors for elderberry cultivars [33,35].

On the other hand, neither the TPC nor the MAC showed significant differences between the samples taken during the three years of the study. 

#### 3.1.2. Phenolic Profile

Supplementary Table S1 shows the phenolic profile (caffeoylquinic acids, anthocyanidins and flavonols) of the 79 analysed samples. Table 2 and Table 3 summarize the values of these compounds in elderberry fruit depending on the harvest year.

One of the characteristics of this fruit is its black–purple colour due to its high anthocyanidin content (Table 2). Seven anthocyanidins derived from the aglycones cyanidin and pelargonidin were quantified in the set of samples analysed. Qualitatively, it is worth highlighting the anthocyanidins cyanidin 3-*O*-sambubioside (C3S), cyanidin 3-*O*-glucoside (C3G), cyanidin 3,5-*O*-diglucoside (C3G5G) and cyanidin 3-*O*-sambubioside-5-*O*-glucoside (C3S5G), which were detected in all the samples analysed, while cyanidin 3-*O*-rutinoside (C3R), pelargonidin 3-*O*-glucoside (P3G) and pelargonidin 3-*O*-malonylglucoside (P3MG) were occasionally detected in a limited group of samples (Supplementary Table S1). From a quantitative point of view, it is worth highlighting the levels of C3S (most concentrated anthocyanidin in 59/79 samples, mean value: 15,932 µg/g of DW, Table 2) and C3G (most concentrated anthocyanidin in 20/79 samples; mean value: 12,393 µg/g of DW), representing between them more than 85% of the total anthocyanin content, in accordance with the results of other authors for this species [5,11]. On the other hand, the total content estimated as a sum via HPLC was in agreement with that obtained for the MAC, with an average HPLC/MAC ratio = 1.12.

Another important group of extractable phenols were flavonols, mainly derived from quercetin and, to a lesser extent, from kaempferol and isorhamnetin. Thus, all the samples analysed presented quercetin 3-*O*-rutinoside as the main flavonol, with a mean value of 4275 µg/g of DW, followed by quercetin 3-*O*-glucoside (mean value: 335 µg/g of DW) and quercetin 3-*O*-acetylglucoside (mean value: 196 µg/g of DW), which together accounted for more than 95% of the extracted flavonols (Table 3). Other flavonols that were detected, but with little interest from a quantitative point of view, were kaempferol 3-*O*-rutinoside, ranging between 18 and 189 µg/g of DW, isorhamnetin 3-*O*-rutinoside (0–129 µg/g of DW), quercetin (0–93 µg/g of DW) and the glucosides of kaempferol and isorhamnetin (0–18 µg/g of DW).

Regarding phenolic acids (Table 2), three caffeoylquinic acid isomers were quantitated: 3-*O*-caffeoylquinic acid (neochlorogenic acid), 4-*O*-caffeoylquinic acid (crypto-chlorogenic acid) and 5-*O*-caffeoylquinic acid (chlorogenic acid), with the latter being the highest in all cases, with values between 506 and 4054 µg/g of DW, followed by neochlorogenic (48–1598 µg/g of DW) and finally crypto-chlorogenic (22–348 µg/g of DW).

In general, the levels of extractable phenolic compounds in elderberry were found to be similar to those described by authors for cultivars and wild individuals of the species [2,5,11,17,18,25,36], whilst highlighting (for their high concentration) the anthocyanins C3S, C3G, C3S5G and quercetin 3-*O*-rutinoside among the flavonols and chlorogenic acid among the caffeoylquinic acids. The contribution of these five compounds accounted for between 85 and 99% of the total estimated phenolic content in the samples collected in this study.

On the other hand, only two minor phenolic compounds (crypto-chlorogenic acid and isorhamnetin-3-O-glucoside), whose joint contribution to the phenolic profile is less than 1%, showed significant differences due to the year of collection. Although it is known that the harvesting year had a stronger influence on the chemical composition than the cultivar [10], the absence of significant differences between the samples collected during each of the three years of study, both for the TPC and MAC, and for the rest of the analytes, provides robustness to the database obtained, since it allows the influence of environmental factors on the phenolic content of the species at a local level to be ruled out. 

#### 3.1.3. Antioxidant Activity

The antioxidant activity of phenolic extracts was evaluated using three complementary methods: the DPPH and ABTS methods determine the radical scavenging activity, and the FRAP method evaluates the reducing power of the extracts. Supplementary Table S1 shows the antioxidant activity of the 79 samples analysed. Table 4 summarizes the values found in the elderberry fruits according to the harvest year.

The results showed mean values of 270.7 µmol of Trolox/g of DW (DPPH assay), 374.7 µmol of Trolox/g of DW (ABTS assay) and 709.9 µmol of Fe^2+^/g of DW (FRAP assay). It should be noted that a high correlation was detected between the values obtained in the DPPH and ABTS assays (r_DPPH-ABTS_ = 0.941), while the correlation, although significant, was notably lower between these methods and FRAP (r_DPPH-FRAP_ = 0.50; r_ABTS-FRAP_ = 0.58). This apparent discrepancy between the results of the methods used to estimate the antioxidant activity can be explained if it is borne in mind that the phenolic compounds present in the extracts are attributed with different levels of antioxidant activity with each of the methods [37], and that these methods are based on different mechanisms (FRAP: single electron transfer; DPPH and ABTS: mixed mechanism of hydrogen atom transfer and single electron transfer) [38]. In a study of elderberry polyphenol content, Ferreira et al. [10] showed that, depending on the levels of the Trolox equivalent antioxidant capacity (TEAC) of the sample, the contribution of the flavonoids is different, pointing out that levels of quercetin 3-*O*-glucoside, crypto-chlorogenic acid and cyanidin-3-sambubioside-5-glucoside contribute positively to high TEAC samples.

In the present study, it was possible to build significant regression models to estimate the antioxidant activities with the three assays (ABTS, DPPH and FRAP) from the components of the phenolic profile of elderberry fruit (Table 5). These models presented differences in the importance of each of the variables that compose them. Thus, while for the ABTS and DPPH assays, the models included up to eight significant variables (six of them the same), on the contrary, the model adjusted to estimate the values of the reducing power only included three significant variables, of which two were not significant in the models for the antiradical activity. In all cases, the variables with the greatest weight (highest standardized coefficients, Table 5) were the major anthocyanins C3G and C3S, highlighting the high negative weight of P3GM in the estimation of antiradical activities.

In addition, from one year to another, there were significant differences in the antioxidant activities of the collected samples, as measured using each of the three methods employed (Table 4). Thus, while the reducing power (FRAP assay) was greater in the collection of samples taken in 2023, the radical scavenging activity (ABTS and DPPH assays) was greater for the set of samples taken in 2021. It is noteworthy that these differences were not detected for the TPC and MAC, with which they are highly correlated, a finding that was also reported by Csorba et al. [35] during the monitoring of elderberry cultivars over a period of 3 years; these authors reported significant differences in reducing power depending on the year of cultivation, but not in the TPC or MAC. Although different explanations have been provided for the differences between harvests for the same cultivar (10, 33), it must be taken into account that the samples collected in this study come from different specimens and years, collected over an area of approximately 2900 km^2^, with different soils, non-homogeneous climatic conditions and a wide range for the harvest period (from 11 July to 17 September), with all of these factors representing possible causes of the differences in the phenolic profiles as well as their contribution to the antioxidant activity (Supplementary Table S1).

### 3.2. Comparison with Other Berries

As a reference to allow for an assessment of the phenolic potential and antioxidant activity of the collected individuals, different berries available in the SERIDA field collection were analysed (a cultivar of blackberry, a cultivar of raspberry and three cultivars of blueberry belonging to the species *V. corymbosum*, *V. virgatum* and a *V. sp.* hybrid) as well as a commercial sample of dried elderberry (Table 6).

Firstly, it stood out that all the elderberry samples presented higher levels of TPC, MAC, FRAP and DPPH than the commercial sample and in 71 of the 79 study samples for the ABTS value, which highlights the interest of local elderberries. Especially relevant was the low MAC content in the commercial sample compared to those of the wild ones, 6.6 times lower than the average content detected for the set of samples, and values between 1.6 times lower for ABTS and 2.2 times for DPPH for antioxidant activities. In this regard, it is known that anthocyanins are especially sensitive to degradation during the drying and storage processes [39,40].

Regarding the rest of the berries, although the number of samples cannot be considered representative, the results allow us to reaffirm the interest of the local elderberries, since the average content of the collected samples was higher in all parameters (TPC, MAC, DDPH, ABTS, FRAP) than that detected in the three samples of *Vaccinium spp.* and raspberry and similar to that found in the blackberry sample.

### 3.3. Effect of Overripening on Phenolics and Antioxidant Properties of Elderberries

The effect of overripening was evaluated in a subset of samples taken from 24 individual trees. Table 7 shows a summary of the results for each of the stages and parameters analysed. 

Several authors have described the maximum anthocyanin content in elderberries at the end of the ripening process, compared to the flavonol content, which remains constant or decreases from the immature state to maturity [14,16,41]. Our results, on the other hand, showed that after the optimal point of maturity, at which the fruits begin to physically deteriorate due to water loss, they do not present significant changes in their antioxidant properties or in most of the phenolic compounds (Table 7), in accordance with Imenšek et al. [13]. Thus, significant differences were only detected in the content of quercetin, which increased significantly. Although the small decrease in quercetin-3-*O*-rutinoside (quercetin 6-*O*-α-l-rhamnosyl-d-glucose) was not significant, the degradation of this molecule, the main flavonol of the fruit, whose decrease is described during ripening [16], could contribute to the significant increase in quercetin through a first stage of release of rhamnose [41] with the subsequent hydrolysis of quercetin-3-*O*-glucoside to generate the aglycone quercetin. However, the increase in quercetin is not of interest from a quantitative point of view as it is a minor compound.

On the contrary, it is especially important to observe that with overripening, there were no changes either in the content of anthocyanins, the main phenolic components of elderberry, or in the antioxidant activity, since these findings allow for the revaluation of a raw material that is, in principle, unsuitable for direct marketing, to obtain these biomolecules of interest in different industrial processes [2,19,24,42].

### 3.4. Microflora in Elderberries

To establish the suitability of the samples for different industrial processes, the microbial content was determined in a set of 24 samples analysed in the previous section at two stages: fully ripe (FR) and overripe (OR). Table 8 shows the counts in the samples analysed.

In the FR samples, the yeast counts ranged between < 2 and 8.0 log_10_ cfu/g, with most samples displaying high levels (7–8 log_10_ cfu/g). On the contrary, the moulds were the microorganisms with the lowest counts, with 7/24 samples showing levels < 100 cfu/g (Table 8). The fungal contamination level of other berries such as blueberries, blackberries, raspberries and strawberries has been associated with the surface type (smooth or bumpy) and hardness of the skin [21]. In our study, the detected fungi contaminate the fruit in the field and start their spoilage on the elder trees. Moreover, it has been described that the biota of fruits is mainly dominated by yeasts and fungi, and yeasts often achieve earlier colonization than fungi due to their faster growth [43], a statement which is supported by our results. 

LAB play a crucial role in the fermentative processes. They are responsible for both malolactic fermentation, a process essential in winemaking and cider production, and the spontaneous lactic acid fermentation of raw vegetables and fruits [43,44]. LAB were found to be present on FR elderberries over a broad range of concentrations (<2.0–7.8 log_10_ cfu/g), with the highest number of samples falling in the 5–6 log_10_ cfu/g range (Table 8).

AAB, along with yeasts and LAB, are part of the autochthonous microflora of the fruits, and high counts of these bacteria are not desired, as they cause the deterioration of the fruit and its juices. The AAB counts were in the range of 3.7 to 8.0 log_10_ cfu/g in the FR samples, with the most frequent values being greater than 5 log_10_ cfu/g. Barbe et al. [45] found a population size in the range of 1 to 5 log_10_ cfu/g in healthy grape berries, while in grapes damaged by *Botrytis cinerea* (grey rot), AAB populations can reach up to 6 log_10_ cfu/g, with *Gluconobacter oxydans* being the most frequently represented species [45]. With this in mind, the levels of AAB in local elderberries should be taken into account. 

The mean count for mesophilic microorganisms in the ripe fruit was around 6.1 log_10_ cfu/g, with a range of 4.0–7.7 log_10_ cfu/g. The results obtained show higher values than those described in elderberry juice [46]. This was the only group of microorganisms that significantly increased its presence in overripe berries, from an average level of 6.1 log_10_ cfu/g in FR berries to 6.9 log_10_ cfu/g in OR berries.

Martins et al. [47] have reported an increase in the bacterial microflora when grape berries become overripe. However, although in our study a trend towards an increase in microorganisms during maturation was observed for all the microbial groups (Supplementary Figures S1–S5), the high variability detected between the samples for the same ripening stage (Table 8), with ranges of more than four orders of magnitude in all cases, does not allow positive conclusions to be drawn in this sense. In any case, it must be noted that the acidic environment of the samples (pH average = 3.6) allows for the growth of LAB, AAB, yeasts and moulds, but is a significant antibacterial factor, since in such acid conditions, most pathogens do not develop [48].

## 4. Conclusions

The study of the fruits of wild elderberry specimens from Asturias (northern Spain) showed a high variability in phenolic compounds similar to or higher than that described for cultivars and specimens located in other locations. The fruits have a high antioxidant activity related to a high anthocyanidin content, especially that of cyanidin-3-O-sambubioside and cyanidin-3-O-glucoside, making them of interest to the agri-food and pharmaceutical industries. Microbiologically, values of yeasts, fungi and bacteria were detected in very variable concentration ranges, so their levels should be monitored in those applications that require strict control, such as juice or wine production. However, even overripe fruits are of interest as a source of anthocyanidins, since their concentration and antioxidant capacity remain after the optimal ripening period, and their use would promote sustainability and a better use of natural resources. The database generated covered the usual harvesting period of this fruit in the area (second half of July–first half of September), with specimens well adapted to the environment, so some of them, with especially high anthocyanidin contents, could be included in breeding programs.

## Figures and Tables

**Table 1:** Total phenolic content (TPC) and monomeric anthocyanin (MAC) in wild elderberry fruit.

Harvest Year		TPC ^1^	MAC ^2^
2021 (n = 31)	mean ± SD	44.8 ± 8.8 ^a^	29.0 ± 8.2 ^a^
	min-max	31.3–66.4	15.5–49.4
2022 (n = 22)	mean ± SD	41.2 ± 10.6 ^a^	23.1 ± 11.7 ^a^
	min-max	28.4–76.1	6.3–58.3
2023 (n = 26)	mean ± SD	44.9 ± 11.9 ^a^	26.5 ± 11.5 ^a^
	min-max	24.0–75.8	9.0–53.7
Total (n = 79)	mean ± SD	43.8 ± 10.4	26.5 ± 10.5
	min-max	24.0–76.1	6.3–58.3

^1^: expressed as mg of gallic acid equivalent/g of dry weight. ^2^: mg of cyanidin 3-O-glucoside/g of dry weight. Different letters in a column mean significant differences between years according to Duncan’s test.

**Table 2:** Caffeoylquinic acids and anthocyanins in wild elderberry fruit.

Harvest Year		Neochlorogenic *	Cryptochlorogenic *	Chlorogenic **	C3G5G **	C3S5G **	C3G **	C3S **	C3R *	P3G *	P3MG *
2021 (n = 31)	mean ± SD	388.5 ± 179.1 ^a^	104.8 ± 35.5 ^b^	1.6 ± 0.9 ^a^	0.2 ± 0.1 ^a^	1.3 ± 0.8 ^a^	14.3 ± 5.7 ^a^	1.6 ± 0.9 ^a^	27.5 ± 152.9 ^a^	0 ± 0 ^a^	16.9 ± 45.7 ^a^
	min-max	47.6–676.2	42.3–165.4	0.5–3.6	0.1–0.4	0.5–3.5	4.9–26.8	0.5–3.6	0–851.1	0–0	0–167.2
2022 (n = 22)	mean ± SD	438.5 ± 338.4 ^a^	164.8 ± 62.5 ^a^	2.0 ± 1.0 ^a^	0.2 ± 0.1 ^a^	1.0 ± 0.4 ^a^	11.6 ± 6.0 ^a^	2.0 ± 1.0 ^a^	0 ± 0 ^a^	40.7 ± 143.6 ^a^	69.4 ± 149.9 ^a^
	min-max	116.2–1597.5	78.4–347.9	0.7–4.0	0.1–0.3	0.3–2.1	3.2–28.5	0.7–4.0	0–0	0–665.8	0–668.4
2023 (n = 26)	mean ± SD	394.3 ± 304.9 ^a^	79.4 ± 55.8 ^b^	1.5 ± 0.8 ^a^	0.2 ± 0.1 ^a^	1.2 ± 0.7 ^a^	10.8 ± 5.8 ^a^	1.5 ± 0.8 ^a^	0 ± 0 ^a^	24.6 ± 78.8 ^a^	75.1 ± 115.7 ^a^
	min-max	68.9–1207.6	21.5–256.8	0.5–4.1	0.1–0.4	0.3–3.0	3.1–22.5	0.5–4.1	0–0	0–365.4	0–487.1
Total (n = 79)	mean ± SD	404.3 ± 271	113.2 ± 60.8	1.7 ± 0.9	0.2 ± 0.1	1.2 ± 0.7	12.4 ± 5.9	1.7 ± 0.9	10.8 ± 95.8	19.4 ± 88.5	50.7 ± 109
	min-max	47.6–1597.5	21.5–347.9	0.5–4.1	0.1–0.4	0.3–3.5	3.1–28.5	0.5–4.1	0–851.1	0–665.8	0–668.4

C3S: cyanidin 3-*O*-sambubioside; C3G: cyanidin 3-*O*-glucoside; C3G5G: cyanidin 3,5-*O*-diglucoside; C3S5G: cyanidin 3-*O*-sambubioside-5-*O*-glucoside; C3R: cyanidin 3-*O*-rutinoside; P3G: pelargonidin 3-*O*-glucoside; P3MG: pelargonidin 3-*O*-malonylglucoside. Different letters in a column mean significant differences between year according to Duncan’s test. *: expressed as µg/g of dry weight. **: expressed as mg/g of dry weight.

**Table 3:** Flavonols in wild elderberry fruit (µg/g of dry weight).

Harvest Year		QG	QR	QAcG	KG	KR	IsoRG	IsoRR	Q
2021 (n = 31)	mean ± SD	336.5 ± 125.5 ^a^	3752.1 ± 1566.0 ^a^	185.9 ± 155 ^a^	3.1 ± 5.0 ^a^	63.6 ± 42.1 ^a^	3.2 ± 5.7 ^a^	31.8 ± 26.4 ^a^	12.4 ± 19.9 ^a^
	min-max	118.2–538.9	1620.4–6786.0	0–668.2	0–12.5	17.9–188.9	0–17.5	0–110.8	0–92.6
2022 (n = 22)	mean ± SD	313.1 ± 96.3 ^a^	4340.5 ± 1681.2 ^a^	190.9 ± 78.0 ^a^	3.4 ± 6.5 ^a^	73.2 ± 31.6 ^a^	0 ± 0 ^b^	41.7 ± 29.5 ^a^	8.6 ± 12.3 ^a^
	min-max	181.2–555.8	1724.7–6821.7	38.8–340.9	0–18.4	29.3–156.7	0–0	0–119.7	0–48.6
2023 (n = 26)	mean ± SD	353.0 ± 184.2 ^a^	4842.5 ± 2210.2 ^a^	213.6 ± 126.2 ^a^	2 ± 4.2 ^a^	65.9 ± 30.4 ^a^	1.3 ± 3.6 ^ab^	44.3 ± 31.7 ^a^	8.7 ± 19.3 ^a^
	min-max	165.2–1106.7	1621.1–11,304.4	52.5–639.3	0–11.6	20.2–126.5	0–13.0	11–128.6	0–70.8
Total (n = 79)	mean ± SD	335.4 ± 140.3	4274.8 ± 1867.4	196.4 ± 127	2.8 ± 5.2	67 ± 35.5	1.7 ± 4.3	38.7 ± 29.3	10.1 ± 17.7
	min-max	118.2–1106.7	1620.4–11,304.4	0–668.2	0–18.4	17.9–188.9	0–17.5	0–128.6	0–92.6

QG: quercetin 3-*O*-glucoside; QR: quercetin 3-*O*-rutinoside; QAcG: quercetin 3-*O*-acetylglucoside; KG: kaempferol 3-*O*-glucoside; KR: kaempferol 3-*O*-rutinoside; IsoRG: isorhamnetin 3-*O*-glucoside; IsoRR: isorhamnetin 3-*O*-rutinoside; Q: quercetin. Different letters in a column mean significant differences between years according to Duncan’s test.

**Table 4:** Antioxidant activities in wild elderberry fruits.

Harvest Year		DPPH ^1^	ABTS ^1^	FRAP ^2^
2021 (n = 31)	mean ± SD	297.9 ± 31.5 ^a^	428.3 ± 82.3 ^a^	671.8 ± 133.5 ^b^
	min–max	239.7–363.2	276.1–602.2	420.4–986.2
2022 (n = 22)	mean ± SD	264.1 ± 43.1 ^b^	355.9 ± 109.8 ^b^	664.8 ± 212.3 ^b^
	min–max	200.3–377.7	210.5–658.1	377.3–1271.8
2023 (n = 26)	mean ± SD	243.9 ± 38.3 ^b^	326.9 ± 100.4 ^b^	793.4 ± 245.5 ^a^
	min–max	184.0–328.1	155.1–584.5	410.2–1487.5
Total (n = 79)	mean ± SD	270.7 ± 43.6	374.7 ± 105.3	709.9 ± 204.4
	min–max	187.9–328.1	170.3–584.5	410.2–1487.5

^1^: expressed as µmol of Trolox equivalent/g of dry weight. ^2^: expressed as µmol of Fe^2+^/g. Different letters in a column mean significant differences between years according to Duncan’s test.

**Table 5:** Standardized coefficients and coefficient of regression (r) obtained to estimate antioxidant activities from the components of the phenolic profile of elderberry fruit.

	DPPH (r = 0.942)	ABTS (r = 0.960)	FRAP (r = 0.810)
Neochlorogenic	−0.086	−0.069	0.063
Cryptochlorogenic	0.140 *	0.128 **	−0.193
Chlorogenic	−0.024	0.001	0.162
C3G5G	−0.074	0.061	0.003
C3S5G	0.276 ***	0.127	−0.007
C3G	0.597 ***	0.379 ***	0.029
C3S	0.261 **	0.402 ***	0.527 **
C3R	0.106 *	0.017	−0.029
P3G	0.092	0.227 **	0.047
P3MG	−0.380 ***	−0.372 ***	0.148
QG	0.023	0.072	0.197
QR	−0.077	−0.082	0.317 *
QAcG	−0.062	−0.102	−0.012
KG	0.178 **	0.155 **	−0.110
KR	0.040	−0.045	−0.249 *
IsoRG	0.121 **	0.199 ***	−0.045
IsoRR	−0.067	−0.068	−0.067
Q	0.085	0.132 **	0.046

C3S: cyanidin 3-*O*-sambubioside; C3G: cyanidin 3-*O*-glucoside; C3G5G: cyanidin 3,5-*O*-diglucoside; C3S5G: cyanidin-3-*O*-sambubioside-5-*O*-glucoside; C3R: cyanidin 3-*O*-rutinoside (C3R); P3G: pelargonidin 3-*O*-glucoside; P3MG: pelargonidin 3-*O*-malonylglucoside; QG: quercetin 3-*O*-glucoside; QR: quercetin 3-rutinoside; QAcG: quercetin 3-acetylglucoside; KG: kaempferol 3-*O*-glucoside; KR: kaempferol 3-rutinoside; IsoRG: isorhamnetin 3-*O*-glucoside; IsoRR: isorhamnetin 3-*O*-rutinoside; Q: quercetin. *: significant at *p* < 0.1; **: significant at *p* < 0.05; ***: significant at *p* < 0.001.

**Table 6:** Total phenolic content (TPC), monomeric anthocyanin content (MAC) and antioxidant activities in commercial dry elderberry and other berries.

	TPC ^1^	MAC ^2^	DPPH ^3^	ABTS ^3^	FRAP ^4^
Commercial elderberry	21.4	4.0	120.9	235.7	326.8
Raspberry	11.5	3.7	117.7	118.1	376.6
Blackberry	32.7	17.0	319.5	417.9	560.4
Blueberry (*V. virgatum*)	13.2	6.1	112.0	138.4	206.6
Blueberry (*V. corymbosum)*	15.5	4.6	119.5	15.00	316.0
Blueberry (*V. sp. hybrid*)	17.2	9.1	122.5	169.9	291.1

^1^: expressed as mg of gallic acid equivalent/g of dry weight. ^2^: mg of cyanidin 3-*O*-glucoside/g of dry weight. ^3^: expressed as µmol of Trolox equivalent/g of dry weight. ^4^: expressed as µmol of Fe^2+^/g.

**Table 7:** Total phenolic content (TPC), monomeric anthocyanin content (MAC), anthocyanins, flavonols, caffeoylquinic acids and antioxidant activities in fully ripe and overripe elderberry fruits (n = 24). Mean ± SD.

	Fully Ripe	Overripe
TPC ^1^	43.7 ± 10.9	50.3 ± 14.3
MAC ^2^	24.9 ± 12.1	25.5 ± 12.8
DPPH ^3^	253.5 ± 47.9	256.0 ± 41.4
ABTS ^3^	340.2 ± 117.3	352.2 ± 119.7
FRAP ^4^	747.0 ± 235.1	802.9 ± 277.2
Neochlorogenic ^5^	365.0 ± 270.7	295.8 ± 230.9
Cryptochlorogenic ^5^	104.1 ± 70.4	87.4 ± 54
Chlorogenic ^5^	1696 ± 1028.7	1659.8 ± 925
C3G5G ^5^	175.2 ± 63.6	171.0 ± 55.9
C3S5G ^5^	1093.2 ± 417.8	1210.1 ± 524.2
C3G ^5^	10,933.9 ± 6594.9	9991.5 ± 5820
C3S ^5^	15,084.9 ± 7624.9	15,578.9 ± 8398.4
C3R ^5^	0 ± 0	0 ± 0
P3G ^5^	33.2 ± 137.4	28.6 ± 140.2
P3MG ^5^	91.5 ± 148.6	82.4 ± 150.9
QG ^5^	307.7 ± 103.5	360.1 ± 116.4
QR ^5^	4167.3 ± 1830.6	3877.3 ± 1353
QAcG ^5^	191.0 ± 90.9	165.9 ± 68.9
KG ^5^	3.4 ± 6.3	5.8 ± 7.6
KR ^5^	69.5 ± 36.1	67.7 ± 24.9
IsoRG ^5^	0.4 ± 2.0	0.8 ± 2.9
IsoRR ^5^	34.6 ± 25.8	32.3 ± 21.2
Q ^5,^*	10.7 ± 17.3	35.2 ± 43.7

C3S: cyanidin 3-*O*-sambubioside; C3G: cyanidin 3-*O*-glucoside; C3G5G: cyanidin 3,5-*O*-diglucoside; C3S5G: cyanidin-3-*O*-sambubioside-5-*O*-glucoside; C3R: cyanidin3-*O*-rutinoside (C3R); P3G: pelargonidin 3-*O*-glucoside; P3MG: pelargonidin 3-*O*-malonylglucoside; QG: quercetin 3-*O*-glucoside; QR: quercetin 3-rutinoside; QAcG: quercetin 3-acetylglucoside; KG: kaempferol 3-*O*-glucoside; KR: kaempferol 3-rutinoside; IsoRG: isorhamnetin 3-*O*-glucoside; IsoRR: isorhamnetin 3-*O*-rutinoside; Q: quercetin. ^1^: expressed as mg of gallic acid equivalent/g of dry weight. ^2^: mg of cyanidin 3-*O*-glucoside/g of dry weight. ^3^: expressed as µmol of Trolox equivalent/g of dry weight. ^4^: expressed as µmol of Fe ^2+^/g of dry weight. ^5^: expressed as µg/g of dry weight. *: significant differences.

**Table 8:** Results of microbiological count in the samples analysed.

		Fully Ripe	Overripe
Aerobic mesophilic	Range ^a^	4.0–7.7	4.3–8.2
Percentage (%) of samples in the indicated interval	<2		
	2–3		
	3–4	8.3	
	4–5	4.2	12.5
	5–6	29.2	8.3
	6–7	37.5	16.7
	7–8	20.8	54.2
	8–9		8.3
Lactic acid bacteria	Range ^a^	<2.0–7.8	<2.0–7.5
Percentage (%) of samples in the indicated interval	<2	20.8	4.2
	2–3	4.2	16.7
	3–4	12.5	8.3
	4–5	8.3	4.2
	5–6	29.2	20.8
	6–7	16.7	20.8
	7–8	8.3	25.0
Acetic acid bacteria	Range ^a^	3.7–8.0	5.1–8.3
Percentage (%) of samples in the indicated interval	< 2		
	2–3		
	3–4	4.2	
	4–5	12.5	
	5–6	16.7	25.0
	6–7	41.7	41.7
	7–8	25.0	29.2
	8–9		4.2
Yeasts	Range ^a^	<2.0–8.0	<2.0–8.5
Percentage (%) of samples in the indicated interval	<2	4.2	4.2
	2–3		
	3–4	16.7	4.2
	4–5		4.2
	5–6	8.3	12.5
	6–7	12.5	4.2
	7–8	58.3	45.8
	8–9		25.0
Moulds	Range ^a^	<2.0–6.0	<2.0–6.5
Percentage (%) of samples in the indicated interval	<2	29.2	33.3
	2–3		
	3–4		
	4–5	41.7	8.3
	5–6	29.2	45.8
	6–7		12.5

^a^: log_10_ cfu/g of fresh elderberries.

## Data Availability

Data are contained within the article.

## Supplementary Materials

The following supporting information can be downloaded at https://www.mdpi.com/article/10.3390/antiox12111986/s1: Supplementary Table S1. Location, sampling date, phenolic composition and antioxidant activity of sampled individuals. Supplementary Figure S1. Aerobic mesophilic counts in elderberry samples. Supplementary Figure S2. Lactic acid bacteria counts in elderberry samples. Supplementary Figure S3. Acetic acid bacteria counts in elderberry samples. Supplementary Figure S4. Yeast counts in elderberry samples. Supplementary Figure S5. Mould counts in elderberry samples.
