# Supplementary figures and images for: The Phenolic Composition, Antioxidant Activity and Microflora of Wild Elderberry in Asturias (Northern Spain): An Untapped Resource of Great Interest
Source: Antioxidants (Basel). 2023 Nov 9;12(11):1986. doi: 10.3390/antiox12111986 (PMC10669248; doi:10.3390/antiox12111986)

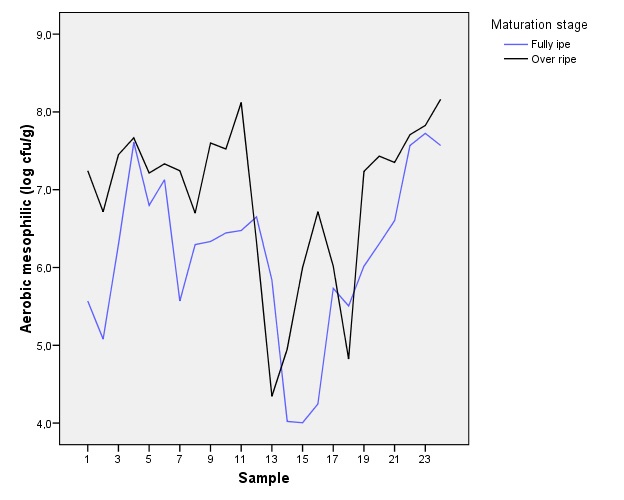

Supplement: Supplementary file 1 [file antioxidants-12-01986-s001.zip › SF-1.JPG]

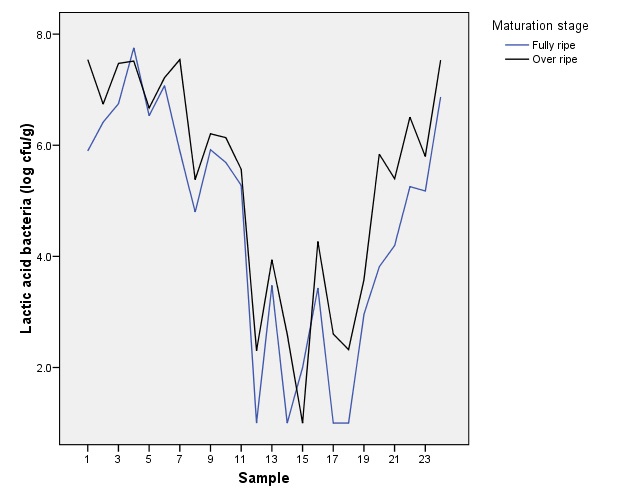

Supplement: Supplementary file 1 [file antioxidants-12-01986-s001.zip › SF-2.JPG]

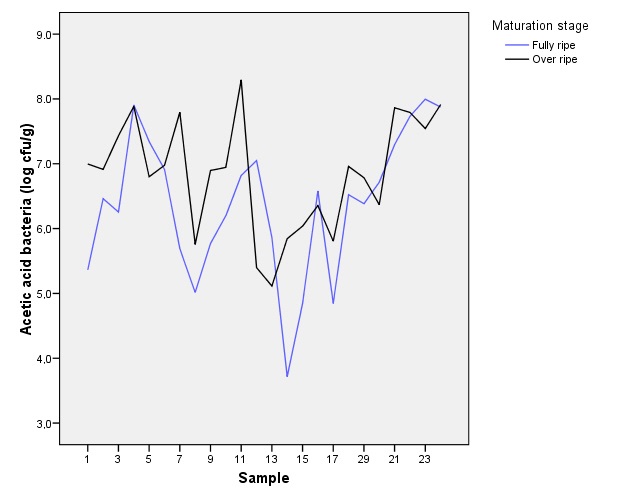

Supplement: Supplementary file 1 [file antioxidants-12-01986-s001.zip › SF-3.JPG]

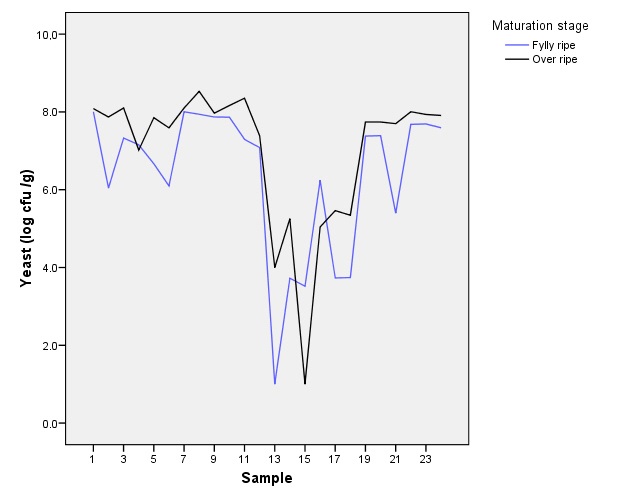

Supplement: Supplementary file 1 [file antioxidants-12-01986-s001.zip › SF-4.JPG]

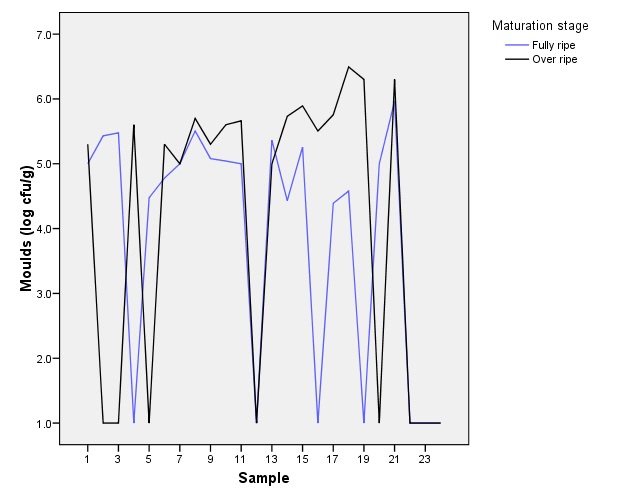

Supplement: Supplementary file 1 [file antioxidants-12-01986-s001.zip › SF-5.JPG]
